# *Momordica charantia* L. Extract Protects Hippocampal Neuronal Cells against PAHs-Induced Neurotoxicity: Possible Active Constituents Include Stigmasterol and Vitamin E

**DOI:** 10.3390/nu13072368

**Published:** 2021-07-10

**Authors:** Nattaporn Pattarachotanant, Anchalee Prasansuklab, Tewin Tencomnao

**Affiliations:** 1 Natural Products for Neuroprotection and Anti-Ageing Research Unit, Chulalongkorn University, Bangkok 10330, Thailand; nat.ahs11@gmail.com; 2 Department of Clinical Chemistry, Faculty of Allied Health Sciences, Chulalongkorn University, Bangkok 10330, Thailand; 3 College of Public Health Sciences, Chulalongkorn University, Bangkok 10330, Thailand

**Keywords:** *Momordica charantia*, polycyclic aromatic hydrocarbons, neurotoxicity, p38 MAPK, p53, cyclin D1, cytochrome P450, stigmasterol, vitamin E, molecular docking

## Abstract

Polycyclic aromatic hydrocarbons (PAHs) have been recognized to cause neurobehavioral dysfunctions and disorder of cognition and behavioral patterns in childhood. *Momordica charantia* L. (MC) has been widely known for its nutraceutical and health-promoting properties. To date, the effect of MC for the prevention and handling of PAHs-induced neurotoxicity has not been reported. In the current study, the neuroprotective effects of MC and its underlying mechanisms were investigated in mouse hippocampal neuronal cell line (HT22); moreover, in silico analysis was performed with the phytochemicals MC to decipher their potential function as neuroprotectants. MC was demonstrated to possess neuroprotective effect by reducing reactive oxygen species’ (ROS’) production and down-regulating cyclin D1, p53, and p38 mitogen-activated protein kinase (MAPK) protein expressions, resulting in the inhibition of cell apoptosis and the normalization of cell cycle progression. Additionally, 28 phytochemicals of MC and their competence on inhibiting cytochrome P450 (CYP: CYP1A1, CYP1A2, and CYP1B1) functions were resolved. In silico analysis of vitamin E and stigmasterol revealed that their binding to either CYP1A1 or CYP1A2 was more efficient than the binding of each positive control (alizarin or purpurin). Together, MC is potentially an interesting neuroprotectant including vitamin E and stigmasterol as probable active components for the prevention for PAHs-induced neurotoxicity.

## 1. Introduction

Polycyclic aromatic hydrocarbons (PAHs) are ubiquitous, persistent environmental pollutants that result from the half-finished burning of coal, oil, tobacco, and garbage. They are integrated into every compartment of daily life as contamination in dietary ingredients, harvested agricultural production, food preparation processes, human activities, and industrial processing. Humans are exposed to the intake of PAHs through several ways including air, water, skin contact, food, and occupational settings. PAHs have been described as causes of toxicity, mutagenicity, and cancer in human [1,2]. Normally, they contain more than two fused aromatic rings, with partial water solubility and high lipophilicity. Their high affinity for lipid-rich tissues might induce several pathological processes in the brain. The acquaintance of prenatal PAHs is related with disorders in childhood including cognition and behavioral patterns [3,4]. PAHs can cause neurotoxicity by inducing apoptosis and cell cycle arrest, the processes requiring many proteins.

Cytochrome P450 (CYP), the enzyme found in the membrane of mitochondria and endoplasmic reticulum, plays critical functions including metabolism of vitamin D and the synthesis of cholesterol and hormones. Additionally, it is likewise related to inducing the metabolism of toxin, drugs, and other endogenous metabolic products and producing intracellular reactive oxygen species (ROS). CYP is divided into many isomers depending on the coded gene. In the current investigation, we studied three prominent isomers of CYP, namely, CYP1A1, CYP1A2, and CYP1B1 responsible for PAHs’ metabolism into toxic metabolites [5,6].

The p38 mitogen-activated protein kinase (MAPK) is a class of MAPK and involved in the stimulation of different biological functions including inflammation, cell apoptosis, cell survival, and cell cycle progression. For cell cycle regulation, p38 can act as a G1/S cell cycle checkpoint by the reduction of cyclin D1 (G1 cyclin regulatory partner). Normally, cyclin D1 can bind to and activate cyclin-dependent kinase 4 and 6 (cdk4/6) for the transition to the S phase and cell cycle progression. There are two independent mechanisms by which p38 regulates cyclin D1, via (1) the suppression of cyclin D1 gene expression and (2) the augmentation of cyclin D1 phosphorylation causing cyclin D1 ubiquitination and proteasomal degradation. The p38 also plays a role in controlling cell survival by activating p53 and inducing cell apoptosis [7,8,9].

*Momordica charantia* L. (MC) belongs to the Cucurbitaceae family and is commonly regarded as bitter cucumber, bitter melon, bitter apple, bitter gourd, and balsam pear. It is extensively used as a food and medicine. In traditional medicine, MC has been indicated in the prevention of hepatic fibrosis and possesses many medicinal properties such as antidiabetic, immunomodulatory, anti-dengue, and antioxidant activities [10,11,12,13,14]. Earlier reports have shown the presence of numerous phytoconstituents, including flavonoids, sterols, and phenolic compounds such as gallic acid, catechin, ascorbic acid, and vitamins in various parts of MC, such as leaves, seeds, and fruits [15].

Presently, air pollution has become a global problem and PAHs are considered among the most dangerous air pollutants that can pose serious threats to humans, particularly brain tissue and neuronal damage. MC is known to possess multipotent pharmacological properties that provide many benefits for human health. However, its neuroprotective role has not yet been elucidated. Therefore, the current study aimed to investigate the protective activity and underlying mechanisms of MC extract against PAHs-induced neuronal damage in HT-22 cells. We also performed the analysis of phytochemical constituents in MC extract and evaluated the ability of those identified compounds to potentially serve as the inhibitors of CYP isomers using an in silico approach.

## 2. Materials and Methods

### 2.1. Chemicals and Reagents

PAHs including phenanthrene (cat#73338), fluoranthene (cat#11474), benzo(a)pyrene (cat#51968), and benzo(b)fluoranthene (cat#30958) were purchased from Sigma-Aldrich (Darmstadt, Germany). The monoclonal rabbit cyclin D1 (92G2, cat#2978), phospho-p38 MAPK (Thr180/Tyr182) (D3F9, cat#4511), p38 MAPK (D13E1, cat#8690), p53 (D2H9O, cat#32532), and GAPDH (14C10, cat#2118) were obtained from Cell Signaling Technology (Beverly, MA, USA).

### 2.2. Plant Extraction

*Momordica charantia* L., designated as MC, was cultivated and harvested during November and December 2020 in Nakornpathom, Thailand (latitude, longitude: 13.838816, 100.050512). Dried, powdered fruits were extracted with absolute ethanol for 1 week followed by filteration and evaporation to dryness. The stock solution of resultant crude extract was prepared in dimethyl sulfoxide (DMSO) and kept at −80 °C for further experiments.

### 2.3. Gas Chromatograph-Mass Spectrometer/Mass Spectrometer (GC-MS/MS) Analysis

GC-MS/MS analysis of MC extract was performed with the facility available at the Scientific and Technological Research Equipment Center (STREC), Chulalongkorn University, Thailand, according to the standard procedure [16]. The mass spectra obtained were correlated with the database from NIST2011 Mass Spectrometry Data Center for the identification of phytoconstituents.

### 2.4. Cell Line

HT-22 cells were a gift from David Schubert (Salk Institute, San Diego, CA, USA). They were maintained in Dulbecco’s Modified Eagle Medium/high glucose (HyClone, Logan, UT, USA) containing 10% Fetal Bovine Serum and antibiotics (100 U/mL penicillin and 100 μg/mL streptomycin) in a humidified atmosphere incubator of 5% CO_2_ at 37 °C.

### 2.5. MTT Assay

The concentrations of PAHs or MC extract were determined for treatment with HT-22. Cells (5 × 10^3^ cells) were treated with MC extracts (0–100 µg/mL) and PAHs (0–5 µg/mL) for 48 h. After the treatment period, MTT (5 mg/mL) was added and incubated for 4 h. After that, 10% sodium dodecyl sulfate (SDS) in 0.01 N HCl was added (overnight), followed by measurement of absorbance at 570 nm. The percentage of cell viability upon PAHs or MC extract treatment was determined as

(1) $$\%\;\mathrm{cell}\;\mathrm{viability}=\frac{\left({\mathrm{Abs}}_{\mathrm{treated}\;\mathrm{cells}}-{\mathrm{Abs}}_{\mathrm{control}}\right)\times 100}{\left({\mathrm{Abs}}_{\mathrm{untreated}\;\mathrm{cells}}-{\mathrm{Abs}}_{\mathrm{control}}\right)}$$

### 2.6. ROS Assay

Cells (5 × 10^3^ cells) were treated or co-treated with MC and phenanthrene (Phe) for 48 h. ROS levels were measured, according to the protocol described elsewhere [16], and the fluorescence was measured using an EnSpire Plate Reader (Perkin-Elmer) with excitation/emission wavelengths of 485/535 nm.

### 2.7. Cell Cycle Assay by Flow Cytometer

Cells (1 × 10^5^ cells) were treated with 5 µg/mL Phe alone or combined with MC for 48 h. After treatment, cells were harvested by trypsinization, followed by washing in cold PBS, centrifugation (400× *g* for 5 min), and suspension (70% or 95% ethanol; 2 h). Further, cells were re-suspended in 1% (*v*/*v*) Triton X-100 in PBS with RNase. Subsequently, PI/Triton X-100 (15 min) was added to the cells and flow cytometry was performed using FACSCalibur (BDbiosciences, San Jose, CA, USA).

### 2.8. Apoptosis Assay by Flow Cytometer

Cells (1 × 10^5^ cells) were co-treated with Phe and MC for 48 h. After treatment, apoptosis was analyzed in each experimental group using flow cytometry (FACSCalibur), according to the standard protocol [16].

### 2.9. Protein Expression by Western Blotting

Treatment to the cells (2.5 × 10^5^ cells) was done, as explained earlier, and proteins from each experimental group were obtained by lysis using NP-40 lysis buffer. Western blotting was performed using the standard experimental procedure [16] with primary antibodies (cyclin D1 (1:2000), phospho-p38 (1:2000), p38 (1:2000), p53 (1:2000), and GAPDH (1:5000)) and anti-rabbit IgG. HRP-linked secondary antibody bands were developed using chemiluminescence detection reagent and the obtained bands were processed using ImageJ software.

### 2.10. Molecular Docking

#### 2.10.1. Ligand Preparation

All chemical structures of the phytochemicals were obtained from PubChem database. We minimized energy using Discovery Studio Visualizer (BIOVIA, San Diego, CA, USA) and converted the file format to the protein databank, partial charge (Q), and atom type (T) or PDBQT using AutoDockTools-1.5.6 software (The Scripps Research Institute, San Diego, CA, USA).

#### 2.10.2. Protein Preparation

The X-ray crystallographic structures of CYP1A1 (PDB ID: 4I8V) [17], CYP1A2 (PDB ID: 2HI4) [18], and CYP1B1 (PDB ID: 3PM0) [19] were retrieved from RCSB Protein Data Bank. Protein structures were processed using the Prepare Protein Set Up in AutoDock tools and converted to PDBQT file format as the inputs for the docking study [20].

#### 2.10.3. Molecular Docking

The docking analyses were performed according to the previous report [20]. In brief, AutoDock 4.2 software package supported by Autodock tools 1.5.6 was used. Lamarckian Genetic Algorithm with default parameters was used to perform the protein–ligand interaction studies and the results were further visualized using the Discovery Studio Visualizer (BIOVIA, San Diego, CA, USA).

### 2.11. Statistical Analysis

The data were represented as mean ± standard deviation (SD) of at least three independent experiments. Statistical significance was analyzed using one-way analysis of variance (ANOVA) followed by a post hoc Tukey test (*p* value < 0.05).

## 3. Results

### 3.1. Effects of the Extract and Polycyclic Aromatic Hydrocarbons (PAHs) on Cell Viability

The MC extract showed a reduction in cell viability in a dose-dependent manner. At 48 h, MC showed 91.44 ± 6.32% and 84.99 ± 6.33% viability for 50 and 100 μg/mL, respectively. The lowest concentrations of MC (5, 10, and 25 µg/mL), with no observed cytotoxicity, were subsequently used for further experiments.

**Figure 1:**
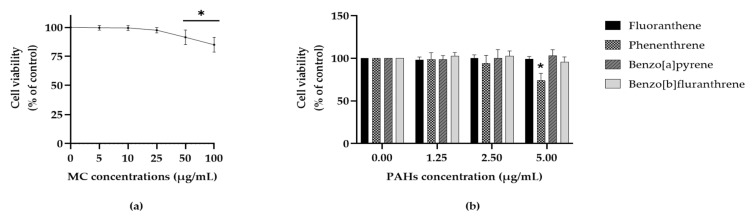
The effect of *Momordica charantia* extract (MC) on cell viability (**a**); the effect of Polycyclic aromatic hydrocarbons (PAHs) on cell viability (**b**); (*n* = 3; * *p* < 0.05 vs. control (0 µg/mL); * *p* values were 0.01 and 0.000 MC-treated groups for concentrations 50 and 100 µg/mL, respectively. For PAHs, * *p* values were 0.002 in 5 µg/mL phenanthrene (Phe)-treated groups.

In the case of Polycyclic Aromatic Hydrocarbons (PAHs) (Figure 1b), the result indicated that the concentration of 5 μg/mL of phenanthrene (Phe) caused significant cytotoxicity (73.75 ± 8.81% viability) was used for further analysis.

### 3.2. Effects of the MC Extract on PAHs-Induced Cytotoxicity and Oxidative Stress

To evaluate the protective effects of MC on PAHs-induced cytotoxicity, cells were treated with 5 μg/mL Phe alone or in combination with different concentrations of MC (5, 10, and 25 μg/mL).

**Figure 2:**
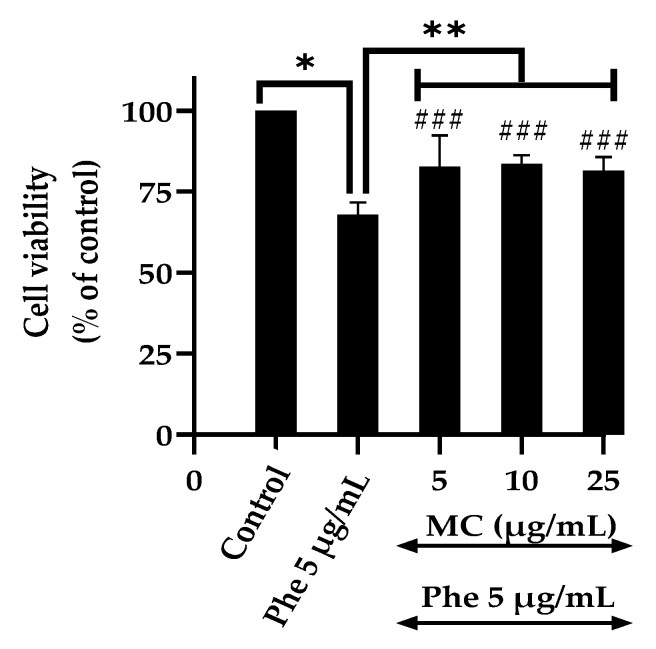
Cytoprotective effect of MC against PAHs-induced neurotoxicity. Cell viability of HT-22 cells treated with 5 µg/mL Phe alone or co-treatment with different concentrations of MC for 48 h; (*n* = 3; * *p* < 0.05 vs. control (0 µg/mL Phe); ** *p* < 0.05 vs. Phe alone; * *p* value was 0.000 in 5 µg/mL Phe alone and ** *p* values were 0.002, 0.001, and 0.006 in Phe combined with 5, 10, and 25 µg/mL MC groups, respectively; ^###^ *p* value was 0.000, 0.001, and 0.000 in Phe group combined with 5, 10, and 25 µg/mL MC compared with control group, respectively.

It was observed that at all the tested concentrations, MC extract was able to protect HT-22 cells from Phe-induced neurotoxicity. Co-treatment with MC showed 82.63% ± 9.68, 83.47% ± 2.76, and 81.34% ± 4.32 cell viability for 5, 10, and 25 μg/mL, respectively, against Phe (Figure 2).

Based on the results, 25 μg/mL MC was used as the working concentration against Phe-induced neurotoxicity models.

**Figure 3:**
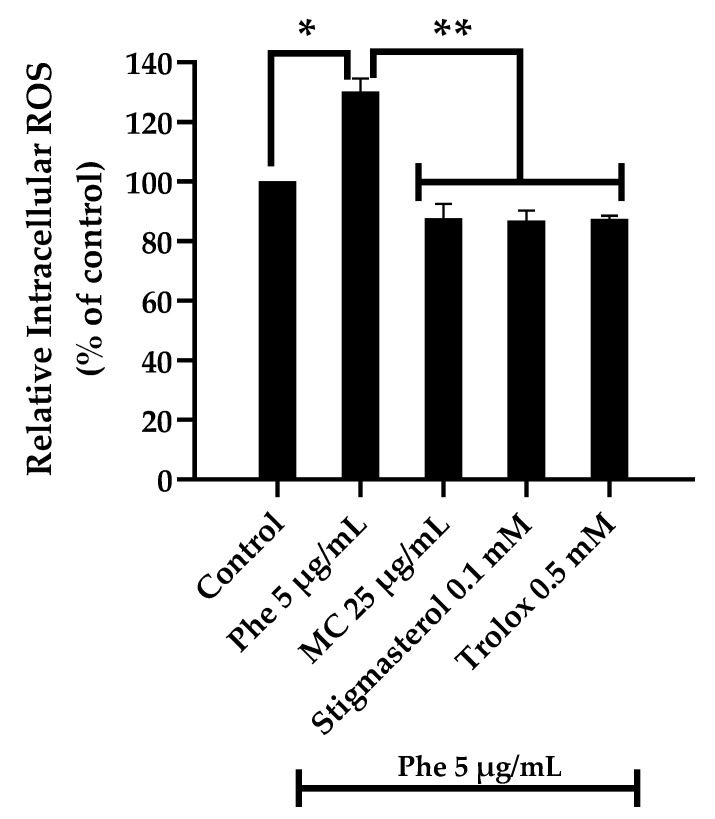
The protective effect of MC on PAHs-induced intracellular ROS formation; * *p* < 0.05 vs. control (0 µg/mL Phe); ** *p* < 0.05 vs. Phe alone; * *p* value was 0.000 in 5 µg/mL Phe alone and ** *p* values were 0.000 in Phe combined with 25 µg/mL MC, 0.1 mM stigmasterol, and 0.5 mM trolox groups. Compared with the control group, there was no significant difference in 25 µg/mL MC, 0.1 mM stigmasterol, and 0.5 mM trolox groups; *n* = 3.

To examine the protective effect of MC extract on Phe-induced ROS generation, HT-22 cells were treated with 5 µg/mL Phe alone or in combination with 25 µg/mL MC. The results showed that the percentage of intracellular ROS (of control) was 130.30 ± 4.40 and 87.70 ± 4.86 in groups treated with Phe alone and combination with 25 µg/mL MC, respectively (Figure 3), indicating MC extract could significantly reduce ROS’ formation.

### 3.3. Effects of MC Extract on PAHs-Induced Apoptosis and Cell Cycle Arrest

**Figure 4:**
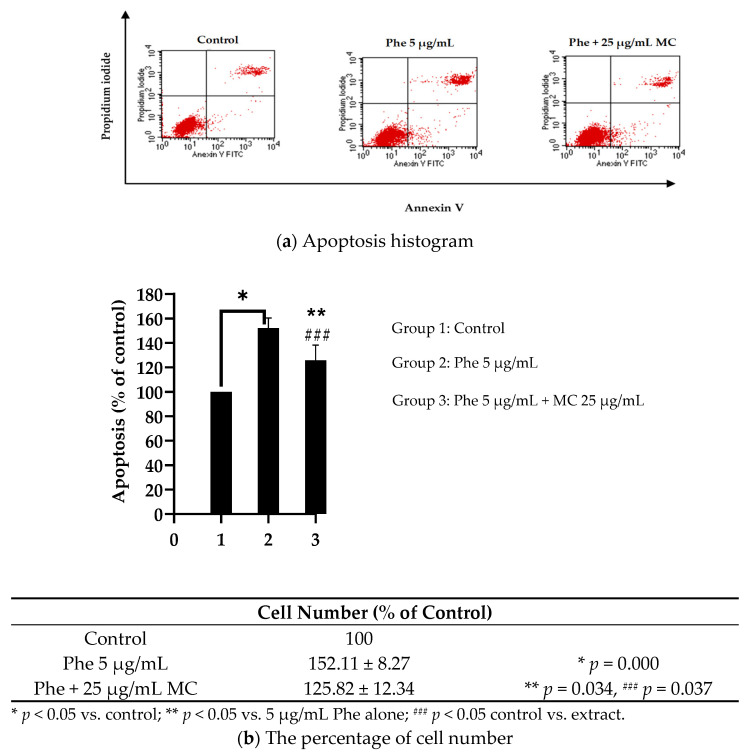
The anti-apoptotic effect of MC against PAHs in HT-22 cells. Apoptotic histograms from each experimental group showed (**a**). Population of apoptotic cells in each group is shown as bar graphs (**b**). * *p* < 0.05 vs. control (0 µg/mL Phe); ** *p* < 0.05 vs. Phe alone. * *p* value was 0.000 in 5 µg/mL Phe alone, ** *p* value was 0.034 in Phe group combined with 25 µg/mL MC, and ^###^ *p* value was 0.037 in Phe group combined with 25 µg/mL MC compared with control group; *n* = 3.

To understand the ability of MC on Phe-induced neurotoxicity, the effect on apoptosis and cell cycle analysis was studied using flow cytometer. The results revealed that the percentage of apoptotic cells treated with 5 µg/mL Phe was significantly higher than in the control group. When co-treated with 25 µg/mL MC, the percentage of apoptotic cells was significantly decreased compared with 5 µg/mL Phe alone (Figure 4).

**Figure 5:**
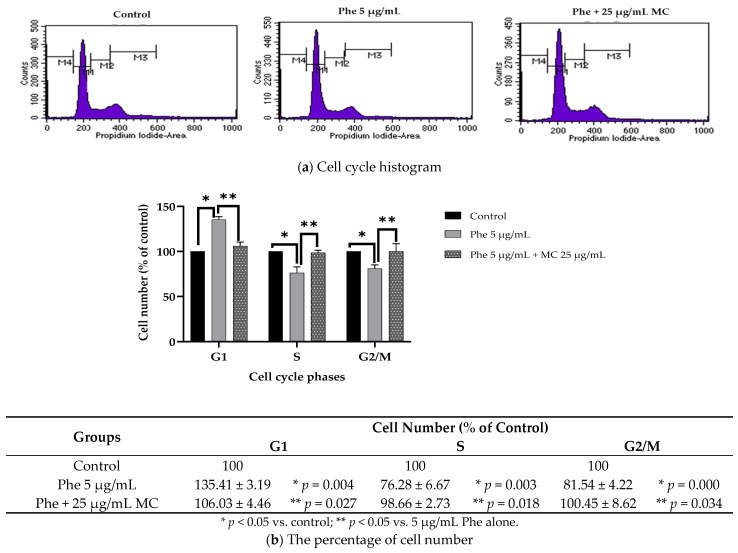
The effect of MC on PAHs-induced cell cycle arrest in HT-22 cells. (**a**) Representative histogram indicating cell cycle arrest, (**b**) cell number (% of control) in experimental group in each phase. * *p* < 0.05 vs. control (0 µg/mL Phe); ** *p* < 0.05 vs. Phe alone. For G1 phase, * *p* value was 0.004 in 5 µg/mL Phe alone, ** *p* value was 0.027 in Phe group combined with 25 µg/mL MC. For S phase, * *p* value was 0.003 in 5 µg/mL Phe alone, ** *p* value was 0.018 in Phe group combined with 25 µg/mL MC. For G2 phase, * *p* value was 0.000 in 5 µg/mL Phe alone, ** *p* value was 0.034 in Phe group combined with 25 µg/mL MC. Compared with the control group, there was no significant difference in 25 µg/mL MC group at all cell cycle phases (*n* = 3).

Furthermore, Phe could induce neurotoxicity by interfering with cell cycle distribution causing cell cycle arrest. Phe (5 µg/mL) could significantly arrest cells at G1 phase and reduce cell distribution at both S and G2/M phases compared to the control group (*p* < 0.05). However, MC treatment could improve cell distribution at all cell cycle phases (Figure 5).

### 3.4. Effects of MC Extract on Apoptotic- and Cell Cycle-Associated Protein Expression

**Figure 6:**
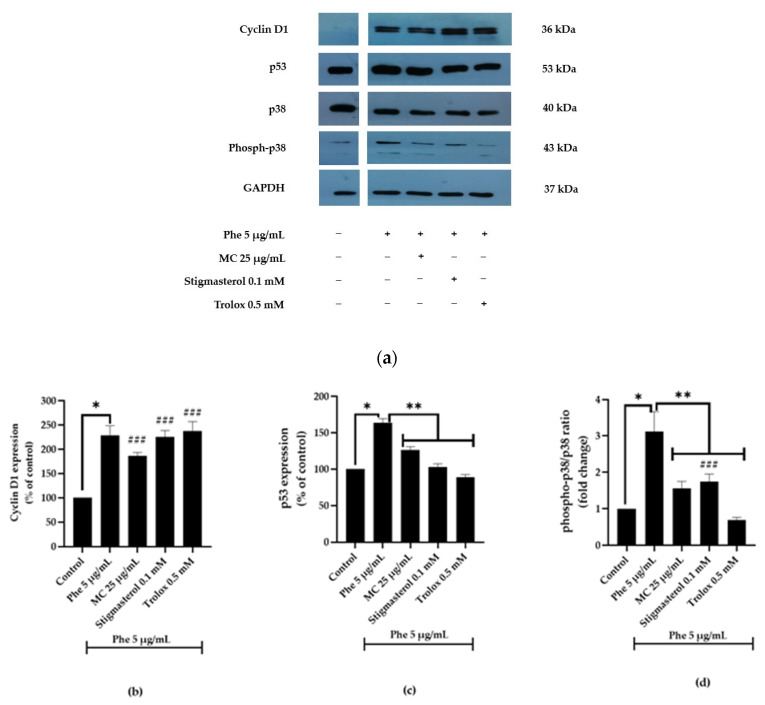
Representative blots showing Cyclin D1, p53, p38, and phospho-p38 expression (**a**). Normalized values of Cyclin D1 (**b**), p53 (**c**), and p38 and phospho-p38 (**d**) against GAPDH. * *p* < 0.05 vs. control; ** *p* < 0.05 vs. 5 µg/mL Phe alone. For Cyclin D1, * *p* = 0.002. For p53, * *p* = 0.002; ** *p* = 0.024, 0.003, and 0.001. For phospho-p38/p38 ratio, * *p* = 0.000; ** *p* = 0.000, 0.001, and 0.000, respectively. For p53 expression, there was no significant difference in all MC-, stigmasterol-, and trolox-treated groups compared with control groups. For cyclin D1 expression, ^###^ *p* value was 0.007, 0.003, and 0.001 in Phe group combined with 25 µg/mL MC, 0.1 mM stigmasterol, and 0.5 mM trolox, respectively, compared with control group. For phospho-p38/p38 ratio, ^###^ *p* value was 0.046 in Phe group combined with 0.1 mM stigmasterol compared with control group (*n* = 3).

Regulation of cell cycle plays a crucial role in the control of neurotoxicity. From flow cytometry analysis, it can be found that Phe causes neurotoxicity by inducing cell apoptosis and affecting cell cycle progression. However, MC extract could reverse the effect of Phe. In addition, the role of p38 in Phe-induced neurotoxicity was assessed with trolox (p38 inhibitor [21]) being used as positive control. Trolox is a well-known, water-soluble vitamin E analogue with reported antioxidant properties against ischemia damage [22,23]. Furthermore, stigmasterol was also used as another positive control because it was one of three major phytochemicals identified by GC-MS/MS. To approve all of these, the expression of four proteins, p38, phospho-p38, p53, and cyclin D1, was studied. The expression of cyclin D1, p53, and phospho-p38/p38 ratio was significantly increased upon treatment with 5 µg/mL Phe alone (* *p* < 0.05 vs. control) (Figure 6). However, after treatment with 25 µg/mL MC, 0.1 mM stigmasterol, or 0.5 mM trolox, both p53 and phospho-p38/p38 ratios were significantly decreased (** *p* < 0.05 vs. 5 µg/mL Phe alone).

### 3.5. Metabolite Profiling of Ethanolic Extract of MC by GC-MS/MS

**Figure 7:**
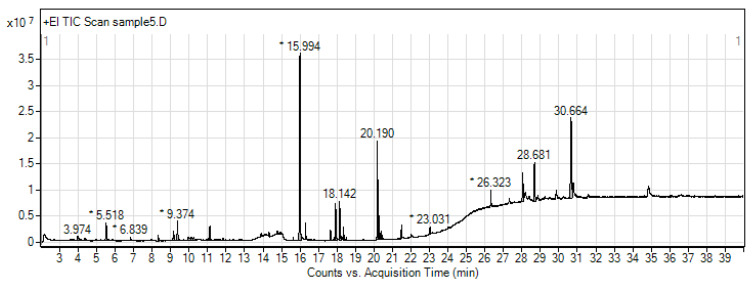
GC-MS/MS chromatogram of *Momordica charantia* L. (MC). * Peaks of proposed phytochemical compounds in MC as suggested by GC-MS/MS.

The GC-MS/MS analysis showed the presence of 28 phytochemicals. Peaks of the phytochemical compounds in MC were shown in Figure 7 with three major constituents being n-Hexadecanoic acid (palmitic acid, 24.13%), stigmasterol (6.88%), and (Z,Z,Z)-9,12,15-octadecatrienoic acid (linolenic acid, 4.60%). Additionally, the other phytochemical constituents present were classified as (1) organic acid (34.4%), mainly composed of fatty acids including myristic acid, palmitic acid, linolenic acid, and stearic acid; (2) phytosterols (11.19%) such as stigmasterol; (3) esters of organic acid (5.04%); (4) phenol (3.5%) including salicylic acid and vitamin E; and (5) other compounds such as coumarins, terpenoid, ketones, amides, aldehydes, and alcohol.

Retention time (RT), molecular formula (MF), molecular weight (MW), nature of compound, and relative concentrations (peak areas %) of these phytochemicals are given in Table 1.

### 3.6. The Ability of MC-Derived Phytochemical Constituents in a Role as the Inhibitors of CYP Isomers Using an In Silico Approach

In the present-day experiment, alizarin and purpurin previously described as strong inhibitors of the activity of all the three CYP isomers were used as the positive control in the molecular docking study [24].

Alizarin exerted the binding energy of −8.63, −8.09, and −8.17 kcal/mol for CYP1A1, CYP1A2, and CYP1B1, respectively (Table 2, Table 3 and Table 4). Furthermore, the binding energy of purpurin to CYP1A1, CYP1A2, and CYP1B1 was −8.84, −7.84, and −8.45 kcal/mol, respectively.

Based on the docking results (Table 2, Table 3 and Table 4), two phytochemicals including vitamin E and stigmasterol showed outstanding inhibition against all of CYP isomers with higher binding energy compared to others.

**Figure 8:**
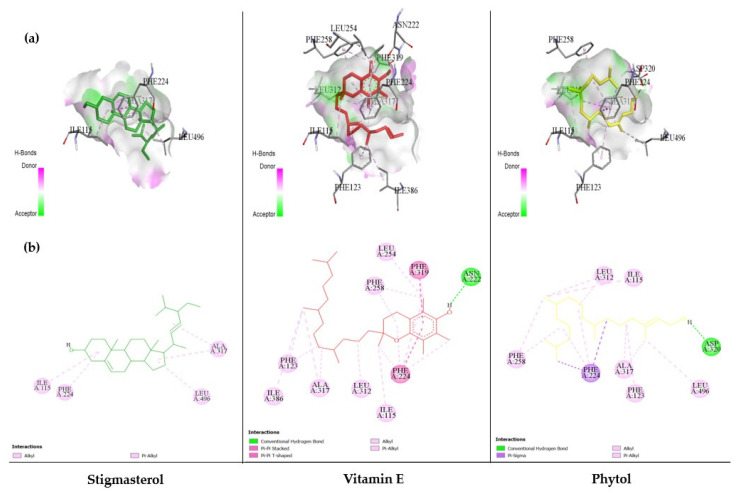
The results of the molecular docking study of CYP1A1 (4I8V) are represented by the 3D diagrams of interaction between each phytochemical and 4I8V (**a**) and by the 2D diagrams of phytochemical–receptor interactions (**b**).

**Figure 9:**
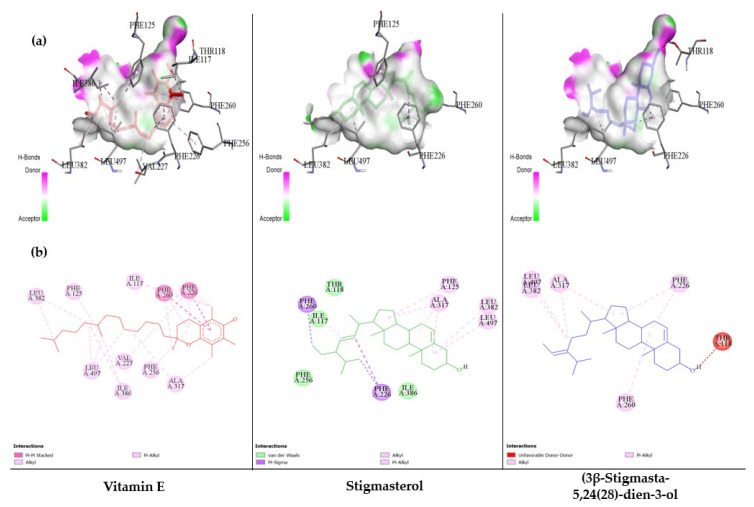
The results of the molecular docking study of CYP1A2 (2HI4) are represented by the 3D diagrams of interaction between each phytochemical and 2HI4 (**a**) and by the 2D diagrams of phytochemical–receptor interactions (**b**).

**Figure 10:**
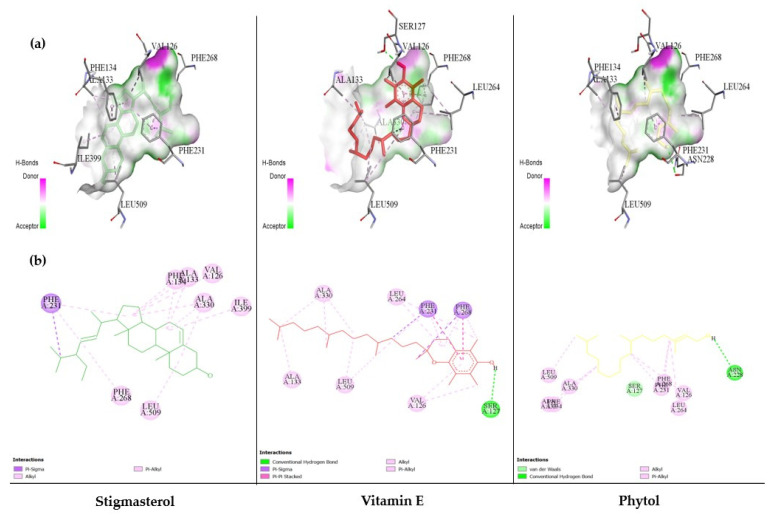
The results of the molecular docking study of CYP1B1 (3PM0) are represented by the 3D diagrams of interaction between each phytochemical and 3PM0 (**a**) and by the 2D diagrams of phytochemical–receptor interactions (**b**).

Noticeably, the energy of both vitamin E binding to CYP1A1 (−8.91 kcal/mol) and CYP1A2 (−8.65 kcal/mol) and stigmasterol binding to CYP1A1 (−8.97 kcal/mol) was higher than both positive controls. The 3D and 2D diagrams of three phytochemicals with higher binding energy are shown in Figure 8, Figure 9 and Figure 10 for CYP1A1, CYP1A2, and CYP1B1, respectively.

## 4. Discussion

*Momordica charantia* L. (MC) is a nutraceutical used in traditional medicine to relieve various ailments and inflammatory diseases including diabetes, cancer, gastric ulcer, fever, high blood pressure, worm infections, malaria, dysentery, and rheumatism therapy [25,26,27]. The metabolite profiling indicated that MC extract contained many functional components, which is consistent with the previous report [15]. It was shown that the bioactive components of MC possess various pharmacological effects including antidiabetic, immunomodulation, neuroprotection, and antitumor properties. This is the first report for MC extract showing the reduction of ROS, anti-apoptotic activity, induction of cell cycle progression, and the expression of apoptosis and cell cycle-checkpoint proteins phospho p38/p38 MAPK, p53, and cyclin D1 against Phe-induced toxicity in HT-22 cells.

Phe is a persistent environmental contaminant belonging to PAHs with applications in the making of resins and pesticides. Phe and PAHs are ubiquitously dispersed in air, landforms, water bodies and enter humans via both occupational and non-occupational exposure by breathing contaminated air, consuming PAHs-contaminated foods, dermal contact, and exposure to smokes from cigarette and open fireplaces [28].

In response to various extracellular stimuli, the p38 MAPK kinase pathway is activated and plays a key role in learning and memory and acts as a vital factor in brain functions [29]. Consequently, deregulation of this pathway may cause neurological disorders.

Interestingly, p38 MAPK responds to various types of cellular stress such as ROS stress and cellular senescence via a series of checkpoints. In addition, p38 mediates G1/S checkpoint through cyclin D1. Normally, binding of cyclin D1 to cyclin-dependent kinase 4 and 6 (Cdk4/6) activates the complex, which is required for the switch to the S phase and cell proliferation. The p38 MAPK keeps a check on cyclin D1 function via phosphorylation, resulting in subsequent degradation. Further, p38 also activate p53 to control cell survival and induce cell apoptosis.

Treatment of Phe significantly increased ROS’ generation and phosphorylation of p38 in HT-22 cells followed by upregulation of p53 and cyclin D1, causing apoptosis and cell cycle arrest.

However, MC significantly decreased p53 and the ratio of phospho-p38/p38 in extract-treated groups except for cyclin D1. Further, the p38 inhibitor trolox was used to confirm that p38 MAPK is a crucial pathway for MC treatment against Phe-induced neurotoxicity. As seen in Figure 6, the expression of all proteins in the MC-treated group was consistent with the expression in trolox-treated group. Noticeably, cyclin D1 expression in both trolox-treated and stigmasterol-treated groups was almost similar to the Phe-treated group, which is in agreement with the previous study reporting that phytosterols could play important roles in anti-cancer activity by increasing cyclin D1 expression causing G1/S phase arrest [30]. The results indicate that the combinatorial effect of stigmasterol and trolox with other phytochemicals in MC extract could probably be more effective than in the form of individual phytochemicals.

The binding affinity of the constituents of MC extract against CYP was analyzed by in silico analysis. For favorable reaction, Gibbs free energy was found to be negative and lessened the binding energy better than the interaction between ligand and protein [31].

These results indicate that both vitamin E and stigmasterol are more potent inhibitors of cytochrome P450 than alizarin and purpurin. In addition, several other phytoconstituents of MC extract also showed interaction indicating the combinatorial activity. The requirement of additional studies is required to verify and confirm the neuroprotective effectiveness of those constituents.

Taken together, our results showed that MC extract could provide a neuroprotective effect against Phe-induced toxicity through p38 MAPK pathway, with vitamin E and stigmasterol as the effective phytochemical constituents.

## 5. Conclusions

Our results reveal that MC is a medicinal plant with antioxidant properties and biologically active constituents. MC could protect cultured neuronal cells (HT22) from PAHs-induced neurotoxicity. The neuroprotective effect of MC is mediated by regulating p38/cyclin D1 checkpoint proteins-dependent cell cycle progression and inhibiting apoptosis. In addition, vitamin E and stigmasterol may be the prominent phytochemicals that, through binding with CYP enzymes, prevent CYP-induced PAHs’ metabolism into toxic metabolites. Nevertheless, the bioactivities of the MC extract need to be additionally studied in a higher-model system, which may provide insight on the mechanism of MC extract against PAHs-induced neuronal cytotoxicity and possibility for the development of novel agents against environmental pollutants.

## Figures and Tables

**Table 1:** Metabolite profiling in *Momordica charantia* L. (MC) obtained by GC-MS/MS analysis.

Compound Name	Nature of Compound	RT (Min)	Area (%)	MF	MW
**Ketone**					
1,2-cyclopentanedione	Lactone	3.649	0.56	C_5_H_6_O_2_	98
d-galactonic acid, γ-lactone	Galactonolactone	14.936	0.16	C_6_H_10_O_6_	178
**Alcohol**					
Glycerin	Polyol compound	3.974	1.09	C_3_H_8_O_3_	92
Phytol	Diterpene alcohol	17.649	1.03	C_20_H_40_O	296
**Aromatic aldehyde**					
Benzeneacetaldehyde		4.976	0.16	C_8_H_8_O	120
**Ester**					
Ethyl hydrogen malonate	Malonic acid ester	5.518	2.77	C_5_H_8_O_4_	132
Hexadecanoic acid, methyl ester	Fatty acid methyl ester	15.632	0.28	C_17_H_34_O_2_	270
Hexadecanoic acid, ethyl ester	Fatty acid ethyl ester	16.303	1.4	C_18_H_36_O_2_	284
(Z,Z,Z)-9,12,15-octadecatrienoic acid, ethyl ester	Fatty acid ethyl ester	18.256	0.39	C_20_H_34_O_2_	306
Octadecanoic acid, ethyl ester	Fatty acid ethyl ester	18.501	0.2	C_20_H_40_O_2_	312
Glycerol 1-palmitate	Monoacylglycerol	21.486	1.2	C_19_H_38_O_4_	330
**Coumarin**					
2,3-dihydro-3,5-dihydroxy-6-methyl-4H-pyran-4-one		6.297	0.09	C_6_H_8_O_4_	144
3,5-dihydroxy-2-methyl-4H-pyran-4-one		6.839	0.35	C_6_H_6_O_4_	142
**Phenol**					
Salicylic acid	Phenolic acid	8.335	0.55	C_7_H_6_O_3_	138
2,4-bis(1,1-dimethylethyl)-phenol	Phenol	11.117	1.17	C_14_H_22_O	206
4-hydroxy-3-methoxy-benzoic acid	Phenolic acid	11.681	0.15	C_8_H_8_O_4_	168
Vitamin E	Tocochromanol	26.323	1.63	C_29_H_50_O_2_	430
**Organic acid**					
5-(hydroxymethyl)-2-furancarboxylic acid	Furoic acid	9.953	1.05	C_6_H_6_O_4_	142
Tetradecanoic acid	Saturated fatty acid	13.894	0.2	C_14_H_28_O_2_	228
n-Hexadecanoic acid	Saturated fatty acid	15.994	24.13	C_16_H_32_O_2_	256
9,12-octadecadienoic acid	Unsaturated fatty acid	17.842	0.33	C_18_H_32_O_2_	280
(Z,Z,Z)-9,12,15-octadecatrienoic acid	Unsaturated fatty acid	17.922	4.6	C_18_H_30_O_2_	278
Octadecanoic acid	Saturated fatty acid	18.142	4.09	C_18_H_36_O_2_	284
**Amide**					
N,N-diethyl-4-methyl-benzamide		11.988	0.07	C_12_H_17_NO	191
Hexadecanamide		18.36	1.24	C_16_H_33_NO	255
**Phytosterol**					
(3β)-Stigmasta-5,24(28)-dien-3-ol		28.046	4.31	C_29_H_48_O	412
Stigmasterol		28.681	6.88	C_29_H_48_O	412
**Terpenoid**					
5-hydroxy-4,7,7-trimethyl-bicyclo[2,2,1]heptan-2-one		14.32	0.36	C_10_H_16_O_2_	168

RT: retention time; MF: molecular formula; MW: molecular weight.

**Table 2:** Docking results of the phytochemical constituents with ^1^ CYP1A1 (^2^ PDB ID: 4I8V).

No.	Compounds/Phytochemical Constituents	Binding Energy (kcal/mol)	Hydrogen Bonding	
			Number	Amino Acid Interaction
	Alizarin (positive control)	−8.63	2	A:SER116:HN–UNK0:O4 :UNK0:H25–:UNK0:O2
	Purpurin (positive control)	−8.84	4	:UNK0:H25–A:ASN255:O :UNK0:H26–:UNK0:O4 :UNK0:H27–A:SER116:OG A:ASP313:CA–:UNK0:O2
1	1,2-cyclopentanedione	−4.50	2	A:SER116:HN–:UNK0:O2 A:ASN255:CA–:UNK0:O1
2	d-galactonic acid, γ-lactone	−4.36	6	A:SER116:HN–:UNK0:O5 :UNK0:H22–A:ASN255:OD1 :UNK0:H21–A:ASN255:OD1 :UNK0:H20–A:ASP313:O A:GLY316:CA–:UNK0:O3 :UNK0:C12–A:ASN255:O
3	Glycerin	−2.61	5	A:SER116:HN–:UNK0:O3 :UNK0:H12–A:ASN255:OD1 :UNK0:H14–A:ASN255:OD1 :UNK0:H13–A:ASN255:OD1 :UNK0:C6–A:SER116:OG
4	Phytol	−8.31	1	:UNK0:H61–A:ASP320:OD1
5	Benzeneacetaldehyde	−5.30	1	A:SER116:HN–:UNK0:O1
6	Ethyl hydrogen malonate	−2.86	4	A:SER116:HN–:UNK0:O4 :UNK0:H17–A:ASN255:O :UNK0:H17–:UNK0:O2 A:ASN255:CA–:UNK0:O2
7	Hexadecanoic acid, methyl ester	−6.47	0	-
8	Hexadecanoic acid, ethyl ester	−6.61	0	-
9	(Z,Z,Z)-9,12,15-octadecatrienoic acid, ethyl ester	−7.90	1	A:SER122:HG–:UNK0:O2
10	Octadecanoic acid, ethyl ester	−6.52	0	-
11	Glycerol 1-palmitate	−5.86	4	A:SER116:HN–:UNK0:O4 :UNK0:H60–A:LEU312:O :UNK0:H61–A:ASN255:OD1 A:GLY316:CA–:UNK0:O2
12	2,3-dihydro-3,5-dihydroxy-6-methyl-4H-pyran-4-one	−5.16	3	A:SER116:HN–:UNK0:O4 :UNK0:H17–A:ASN255:OD1 :UNK0:H18–A:SER116:OG
13	3,5-dihydroxy-2-methyl-4H-pyran-4-one	−4.86	4	A:SER116:HN–:UNK0:O4 :UNK0:H15–A:SER116:OG :UNK0:H16–A:ASN255:OD1 A:GLY316:CA–:UNK0:O1
14	Salicylic acid	−4.42	3	A:SER116:HN–:UNK0:O2 :UNK0:H15–A:SER116:OG :UNK0:H16–A:SER116:OG
15	2,4-bis(1,1-dimethylethyl)-phenol	−7.03	0	-
16	4-hydroxy-3-methoxy-benzoic acid	−4.93	4	A:SER116:HN–:UNK0:O2 :UNK0:H20–A:LEU312:O :UNK0:H19–A:ASN255:OD1 A:ASN255:CA–:UNK0:O1
17	Vitamin E	−8.91	1	:UNK0:H81–A:ASN222:OD1
18	5-(hydroxymethyl)-2-furancarboxylic acid	−3.63	2	A:SER116:HN–:UNK0:O3 :UNK0:H16–A:ASN255:OD1
19	Tetradecanoic acid	−5.41	1	:UNK0:H44–A:ASP313:O
20	n-Hexadecanoic acid (Palmitic Acid)	−5.63	1	:UNK0:H50–A:ASP313:O
21	9,12-octadecadienoic acid	−6.12	2	A:THR497:HN–:UNK0:O2 A:GLY225:CA–:UNK0:O2
22	(Z,Z,Z)-9,12,15-octadecatrienoic acid	−6.76	0	-
23	Octadecanoic acid	−5.93	3	A:SER116:HN–:UNK0:O2 :UNK0:H56–A:ASN255:O :UNK0:H56–A:ASN255:OD1
24	N,N-diethyl-4-methyl-benzamide	−6.81	1	:UNK0:C3–A:GLY316:O
25	Hexadecanamide	−6.83	1	:UNK0:H51–A:ASP320:OD1
26	(3β)-Stigmasta-5,24(28)-dien-3-ol	−4.73	1	:UNK0:H67–A:SER116:OG
27	Stigmasterol	−8.97	0	-
28	5-hydroxy-4,7,7-trimethyl-bicyclo[2,2,1]heptan-2-one	−5.72	1	:UNK0:H28–A:ASN255:OD1

^1^ CYP1A1: cytochrome P450 family 1 subfamily A member 1; ^2^ PDB ID: protein databank identity document.

**Table 3:** Docking results of the phytochemical constituents with ^1^ CYP1A2 (^2^ PDB ID: 2HI4).

No.	Compound	Binding Energy (kcal/mol)	Hydrogen Bonding	
			Number	Amino Acid Interaction
	Alizarin (positive control)	−8.09	2	:UNK0:H25–A:ASP313:OD1 :UNK0:H26–A:ASP313:OD1
	Purpurin (positive control)	−7.84	3	A:THR124:HG1–:UNK0:O4 :UNK0:H26–:UNK0:O4 :UNK0:H27–A:ASP320:OD1
1	1,2-cyclopentanedione	−4.15	3	A:THR124:HG1–:UNK0:O2 A:GLY316:HN–:UNK0:O1 A:ALA317:HN–:UNK0:O1
2	d-galactonic acid, γ-lactone	−3.71	5	A:THR124:HG1–:UNK0:O3 A:ALA317:HN–:UNK0:O2 :UNK0:H21–A:ASN312:O :UNK0:H19–A:ASN312:O :UNK0:H20–A:ASP313:O
3	Glycerin	−2.53	6	A:THR124:HG1–:UNK0:O2 A:ALA317:HN–:UNK0:O1 :UNK0:H12–A:ASN312:O :UNK0:H14–A:ASN312:O :UNK0:H13–A:ASP313:O :UNK0:H13–:UNK0:O1
4	Phytol	−6.82	1	:UNK0:H61–A:GLY316:O
5	Benzeneacetaldehyde	−4.77	2	A:THR124:HG1–:UNK0:O1 :UNK0:C9–A:ASP313:O
6	Ethyl hydrogen malonate	−3.05	3	A:ALA317:HN–:UNK0:O2 :UNK0:H17–A:ASP313:O :UNK0:H17–:UNK0:O2
7	Hexadecanoic acid, methyl ester	−6.00	0	-
8	Hexadecanoic acid, ethyl ester	−6.41	0	-
9	(Z,Z,Z)-9,12,15-octadecatrienoic acid, ethyl ester	−6.64	0	-
10	Octadecanoic acid, ethyl ester	−6.42	1	:UNK0:C21–A:ASP313:OD1
11	Glycerol 1-palmitate	−4.89	0	-
12	2,3-dihydro-3,5-dihydroxy-6-methyl-4H-pyran-4-one	−4.15	3	A:SER122:HG–:UNK0:O2 A:THR124:HG1–:UNK0:O4 :UNK0:H17–A:ASP313:OD1
13	3,5-dihydroxy-2-methyl-4H-pyran-4-one	−3.91	2	A:THR124:HG1–:UNK0:O4 :UNK0:H15–A:ASP313:OD1
14	Salicylic acid	−4.46	3	A:SER122:HG–:UNK0:O1 :UNK0:H15–A:ASP313:OD1 :UNK0:H16–A:ASP313:OD1
15	2,4-bis(1,1-dimethylethyl)-phenol	−7.11	0	-
16	4-hydroxy-3-methoxy-benzoic acid	−4.57	2	:UNK0:H20–A:ASP313:O A:GLY316:CA–:UNK0:O1
17	Vitamin E	−8.65	0	-
18	5-(hydroxymethyl)-2-furancarboxylic acid	−3.49	2	A:THR124:HG1–:UNK0:O3 :UNK0:H16–A:ASP313:O
19	Tetradecanoic acid	−5.37	2	A:THR124:HG1–:UNK0:O1 :UNK0:H44–A:ASP313:O
20	n-Hexadecanoic acid (Palmitic Acid)	−5.34	0	-
21	9,12-octadecadienoic acid	−6.22	1	A:THR124:HG1–:UNK0:O2
22	(Z,Z,Z)-9,12,15-octadecatrienoic acid	−6.06	0	-
23	Octadecanoic acid	−5.48	0	-
24	N,N-diethyl-4-methyl-benzamide	−6.39	0	-
25	Hexadecanamide	−6.23	2	:UNK0:H50–A:ASP313:OD1 A:SER122:CB–:UNK0:O1
26	(3β)-Stigmasta-5,24(28)-dien-3-ol	−7.61	0	-
27	Stigmasterol	−7.82	0	-
28	5-hydroxy-4,7,7-trimethyl-bicyclo[2,2,1]heptan-2-one	−5.78	1	:UNK0:H28–A:ASP313:OD1

^1^ CYP1A2: cytochrome P450 family 1 subfamily A member 2; ^2^ PDB ID: protein databank identity document.

**Table 4:** Docking results of the compounds with ^1^ CYP1B1 (^2^ PDB ID: 3PM0).

No.	Compound	Binding Energy (kcal/mol)	Hydrogen Bonding	
			Number	Amino Acid Interaction
	Alizarin (positive control)	−8.17	3	:UNK0:H25–A:GLY329:O :UNK0:H25–:UNK0:O2 :UNK0:H26–A:ASN228:OD1
	Purpurin (positive control)	−8.45	5	:UNK0:H25–A:GLY329:O :UNK0:H25–:UNK0:O3 :UNK0:H26–A:ASN265:OD1 :UNK0:H26–:UNK0:O4 :UNK0:H27–A:ASN228:OD1
1	1,2-cyclopentanedione	−4.29	2	A:SER127:HN–:UNK0:O2 A:ASN265:CA–:UNK0:O1
2	d-galactonic acid, γ-lactone	−4.24	6	A:SER127:HN–:UNK0:O5 :UNK0:H22–A:ASN265:OD1 :UNK0:H21–A:ASN265:OD1 :UNK0:H19–:UNK0:O4 :UNK0:H20–A:ASN228:OD1 :UNK0:H19–A:PHE231
3	Glycerin	−2.38	5	A:SER127:HN–:UNK0:O3 :UNK0:H12–A:ASN265:OD1 :UNK0:H14–A:ASN265:OD1 :UNK0:H13–A:ASN265:OD1 :UNK0:C6–A:SER127:OG
4	Phytol	−7.13	1	:UNK0:H61–A:ASN228:O
5	Benzeneacetaldehyde	−4.95	1	A:GLN332:HE21–:UNK0:O1
6	Ethyl hydrogen malonate	−2.65	3	A:SER127:HN–:UNK0:O4 :UNK0:H17–A:ASN265:OD1 :UNK0:H17–:UNK0:O1
7	Hexadecanoic acid, methyl ester	−5.69	0	-
8	Hexadecanoic acid, ethyl ester	−6.18	1	A:GLN332:HE21–:UNK0:O1
9	(Z,Z,Z)-9,12,15-octadecatrienoic acid, ethyl ester	−6.87	2	A:GLN332:HE21–:UNK0:O1 :UNK0:C15–A:ASN228:OD1
10	Octadecanoic acid, ethyl ester	−5.80	0	-
11	Glycerol 1-palmitate	−5.24	4	A:SER127:HN–:UNK0:O4 :UNK0:H60–:UNK0:O2 :UNK0:H61–A:ASN265:OD1 A:GLY329:CA–:UNK0:O1
12	2,3-dihydro-3,5-dihydroxy-6-methyl-4H-pyran-4-one	−4.89	3	A:SER127:HN–:UNK0:O4 :UNK0:H17–A:ASN265:OD1 :UNK0:H18–A:SER127:OG
13	3,5-dihydroxy-2-methyl-4H-pyran-4-one	−4.60	3	A:GLN332:HE21–:UNK0:O4 :UNK0:H15–A:ASN228:OD1 :UNK0:H16–A:ASP333:OD2
14	Salicylic acid	−4.22	3	A:SER127:HN–:UNK0:O3 :UNK0:H15–A:ASN265:OD1 :UNK0:H16–A:ASN265:OD1
15	2,4-bis(1,1-dimethylethyl)-phenol	−6.93	1	:UNK0:H37–A:ASP326:OD2
16	4-hydroxy-3-methoxy-benzoic acid	−4.43	3	A:SER127:HN–:UNK0:O4 :UNK0:H17–A:ASN265:OD1 :UNK0:H18–A:SER127:OG
17	Vitamin E	−7.15	1	:UNK0:H81–A:SER127:OG
18	5-(hydroxymethyl)-2-furancarboxylic acid	−3.44	2	:UNK0:H16–A:ASP326:O :UNK0:H15–A:GLY329:O
19	Tetradecanoic acid	−4.81	0	-
20	n-Hexadecanoic acid (Palmitic Acid)	−5.15	0	-
21	9,12-octadecadienoic acid	−6.20	0	-
22	(Z,Z,Z)-9,12,15-octadecatrienoic acid	−5.76	0	-
23	Octadecanoic acid	−5.33	2	A:SER127:HN–:UNK0:O1 :UNK0:H56–A:ASN265:O
24	N,N-diethyl-4-methyl-benzamide	−6.54	1	A:GLN332:HE21–:UNK0:O1
25	Hexadecanamide	−6.03	2	:UNK0:H50–A:ALA330:O :UNK0:H51–A:ASP333:OD2
26	(3β)-Stigmasta-5,24(28)-dien-3-ol	−3.05	1	:UNK0:C22–A:ASN265:OD1
27	Stigmasterol	−8.06	0	-
28	5-hydroxy-4,7,7-trimethyl-bicyclo[2,2,1]heptan-2-one	−4.19	1	A:SER127:HN–:UNK0:O2

^1^ CYP1B1: cytochrome P450 family 1 subfamily B member 1; ^2^ PDB ID: protein databank identity document.

## Data Availability

Not applicable.
